# Alterations of functional connectivity in auditory and sensorimotor neural networks: A case report in a patient with cortical deafness after bilateral putaminal hemorrhagic stroke

**DOI:** 10.1097/MD.0000000000024302

**Published:** 2021-01-22

**Authors:** Dae-Won Gwak, Eunhee Park, Jin-Su Park, Eunji Kim, Min-Gu Kang, Ae-Ryoung Kim, Jae-Eun Lee, Seung-Hwan Jung, Jae-Gyeong Jeong, Kyu-Yup Lee, Yongmin Chang, Tae-Du Jung

**Affiliations:** a Department of Rehabilitation Medicine, Kyungpook National University Chilgok Hospital; b Department of Rehabilitation Medicine, School of Medicine; c Department of Medical & Biological Engineering, Kyungpook National University, Daegu; d Department of Physical Medicine and Rehabilitation, Dong-A University College of Medicine, Busan; e Department of Rehabilitation Medicine; f Department of Otolaryngology; g Department of Radiology, Kyungpook National University Hospital; h Department of Molecular Medicine, School of Medicine, Kyungpook National University, Daegu, Republic of Korea.

**Keywords:** auditory cortex, auditory pathways, diffuse tensor imaging

## Abstract

**Rationale:**

Cortical deafness is a rare auditory dysfunction caused by damage to brain auditory networks. The aim was to report alterations of functional connectivity in intrinsic auditory, motor, and sensory networks in a cortical deafness patient.

**Patient concerns:**

A 41-year-old woman suffered a right putaminal hemorrhage. Eight years earlier, she had suffered a left putaminal hemorrhage and had minimal sequelae. She had quadriparesis, imbalance, hypoesthesia, and complete hearing loss.

**Diagnoses:**

She was diagnosed with cortical deafness. After 6 months, resting-state functional magnetic resonance imaging (rs-fMRI) and diffuse tensor imaging (DTI) were performed. DTI revealed that the acoustic radiation was disrupted while the corticospinal tract and somatosensory track were intact using deterministic tracking methods. Furthermore, the patient showed decreased functional connectivity between auditory and sensorimotor networks.

**Interventions:**

The patient underwent in-patient stroke rehabilitation therapy for 2 months.

**Outcomes:**

Gait function and ability for activities of daily living were improved. However, complete hearing impairment persisted in 6 months after bilateral putaminal hemorrhagic stroke.

**Lessons:**

Our case report seems to suggest that functional alterations of spontaneous neuronal activity in auditory and sensorimotor networks are related to motor and sensory impairments in a patient with cortical deafness.

## Abstract 2

Supplemental Digital Content is available in the text

## 1 Introduction

Cortical deafness is a rare type of auditory dysfunction and is caused by damage to bilateral primary/secondary auditory cortices or subcortical interruption of acoustic radiations.^[1,2]^ A recent case study using diffuse tension imaging (DTI) reported that a patient with bilateral putaminal hemorrhage with cortical deafness showed structural impairment in bilateral acoustic radiations.^[3]^ However, there has been no combined DTI and resting-state fMRI (rs-fMRI) study to investigate both structural and functional disruptions of auditory networks in patients with cortical deafness following bilateral subcortical hemorrhage.

In this study, we present a case of alterations in functional connectivity between intrinsic auditory and sensorimotor networks in a patient with cortical deafness after bilateral putaminal hemorrhage using both DTI and rs-fMRI. In particular, we suggest that these functional alterations may be related to motor and sensory impairments.

The patient and her sister in this case report signed the consent form for publication. And, our study was approved by the Institutional Review Board of Kyungpook National University Chilgok Hospital (2019-06-021).

## 2 Case presentation

**Figure 1:**
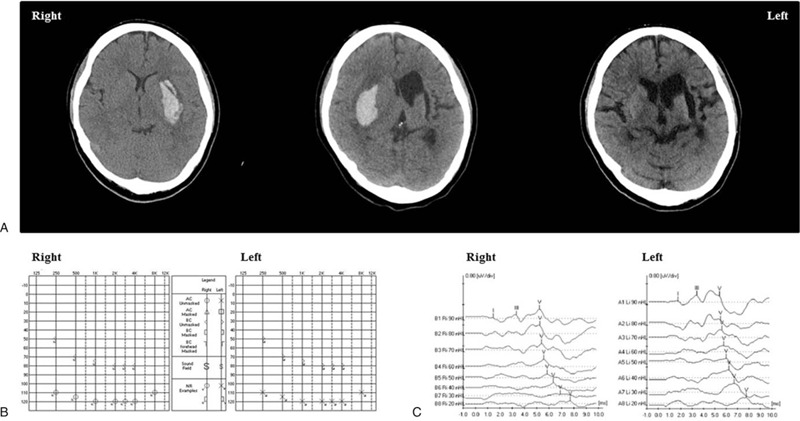
Brain CT were shown in (A) at immediately after the first hemorrhagic stroke (left panel), immediately after the second hemorrhagic stroke (middle panel) and 6 mo after the second hemorrhagic stroke (right panel). The results of pure tone audiometry and brainstem auditory evoked potential test were shown in (B) and (C), respectively.

The case patient was a 41-year-old woman who was an office worker. When she was 34, the first intracranial hemorrhage (ICH) on her left putamen occurred. She had functionally recovered without sequelae in 4 months after the first stroke. She had been diagnosed with hypertension but did not take anti-hypertensive medicines. In January 2018, she visited our hospital emergency room with loss of consciousness. The second ICH on her right putamen was observed in brain computed tomography (CT, Fig. 1A). After conservative management for ICH, the patient was transferred to the Department of Rehabilitation Medicine. At that time, she suffered from quadriparesis, gait disturbance, hypoesthesia, and complete hearing loss. She could not hear any sounds but could speak and understand conversations through lip reading. Communication was mainly done through writing. The pure tone audiometry (PTA) revealed no reliable responses to all frequency (Fig. 1B). The speech reception threshold (SRT) and speech discrimination threshold (SDT) also showed no response. However, the brainstem auditory evoked potential test (BAEP) was observed within normal range of inter-latencies between wave I and III and between wave III and V (Fig. 1C). In addition, the tympanometry, stapedial reflex, distortion product otoacoustic emission (DPOAE) and transient evoked otoacoustic emission (TEOAE) were intact. Her cognitive function was intact. Her Mini Mental State Examination (MMSE) was 27, and the loss of points was caused by disability to perform repetition of language due to hearing loss. Therefore, she was diagnosed with cortical deafness excluding auditory agnosia, aphasia, or cognitive deficit.^[2]^ She was able to walk using a walker but needed intermittent support of a physiotherapist. The level of Functional Ambulation Category (FAC) was 2, and the score of Balance Berg Scale (BBS) was 23. In addition, she needed maximal help performing all categories of activities of daily living, and the score of Korean Modified Barthel Index (K-MBI) was 14. Hypoesthesia in bilateral upper and lower limbs was observed in deep touch and vibration modalities through large sensory nerve fibers and in pain and temperature modalities through small sensory nerve fibers using the current perception test.

The patient took medicines, including anti-hypertensive agents, neurostimulation agents, and antidepressants. In addition, she received conventional physical therapy and occupational therapy for 2 months in accordance with clinical practices guideline for stroke rehabilitation in Korea.^[4]^ Gait and balance training and strengthening exercise of lower limbs was performed for 30 minutes, 2 times a day. Strengthening exercise of upper limbs and training in activity of daily living was performed for 30 minutes, twice a day.

After 6 months following the second hemorrhagic stroke, we performed clinical and neuroimaging assessments. She experienced progressive motor and sensory recovery but disability of complete hearing loss persisted. Follow-up evaluations were performed, and the PTA showed no response to all frequency and the BAEP was still normal. She could walk independently on level ground using a walker, and the level of FAC was 4. Her balance function improved, and the BBS score was 41. She needed minimal to moderate help performing activities of daily living, and the score of K-MBI was 52. In addition, she reported more improvements in feeling sense of deep touch and hot/cold temperature than 6 months ago.

The DTI, rs-fMRI images, and T1-weighted images were obtained using 3T scanner, GE Signa Hdxt for the patient and GE Discovery MR750W for 1 age-matched healthy control. A DTI image using a total of 25 diffusion sampling directions with b-value 1000 s/mm^2^ was acquired. The deterministic tractography was conducted using DSI Studio (http://dsi-studio.labsolver.org) with manually drawn regions of interest (ROIs) based on AAL and HCP 842 tractography atlases. The corticospinal tract was tracked using anterior pons as the seed ROI and primary motor cortex as the terminate ROI. The somatosensory tract was tracked using posterior pons as the seed ROI and primary somatosensory cortex as the terminated ROI. The acoustic radiation was tracked using acoustic radiation as the seed ROI and Heschle gyrus as the terminated ROI (Supplemental table S1). For the rs-fMRI scan, 240 volumes of images were acquired with TR = 2000 ms, TE = 30 and 40 ms, slice thickness = 4 mm, and acquisition matrix = 64 x 64. The rs-fMRI data were preprocessed by Oxford Centre for Functional MRI of Brain's Software Library (FSL) toolbox (https://fsl.fmrib.ox.ac.uk/fsl/fslwiki/). Using FEAT (part of FSL), the following steps were performed: motion correction (MCFLIRT), brain extraction (BET2), temporal filtering, spatial smoothing (FWHM 5 mm). Spatial transformation, linear transformation (FLIRT), and nonlinear transformation (FNIRT) were performed. In Montreal Neurological Institute (MNI) 152 template, cortical ROIs of auditory, sensory, and motor networks were drawn with spheres of 5 mm radius centered in each hemisphere as follows: the primary auditory cortex, the auditory association cortex, the primary somatosensory cortex, the secondary somatosensory association cortex, the multimodal sensory association cortex, the primary motor cortex, and the motor supplementary cortex (Supplemental table S2).

**Figure 2:**
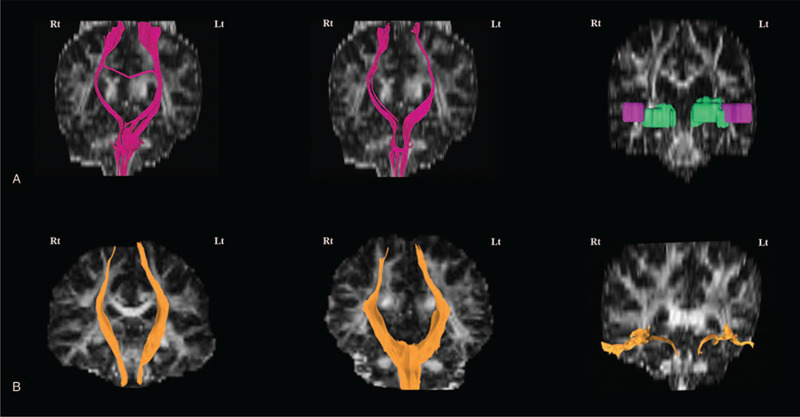
Reconstruction of the corticospinal tract, the somatosensory tract, and acoustic radiation in a patient (A, pink color) and a healthy control (B, orange color) using deterministic tractography. A left column indicated a corticospinal tract, a middle column indicated a somatosensory tract, and a right column indicated an acoustic radiation. A patient's acoustic radiation showed complete absent from Heschl gyrus ROI (pink color) to acoustic radiation ROI (green color).

Motor function and hypoesthesia got better 6 months after the second stroke. From DTI measurement, the mean of fractional anisotropy (FA) value in the corticospinal tract was 0.58 on the left side and 0.60 on the right side (Fig. 2A). In addition, the mean of FA value was 0.56 on the left side and 0.56 on the right side in the somatosensory tract (Fig. 2A). However, bilateral acoustic radiations were not visualized via tractography in a patient. In Figure 2B, corticospinal tract, somatosensory tract, and acoustic radiation were shown in a healthy control. Table 1 provided more detail information about DTI parameters in a healthy control and a patient.

**Table 1:** Diffuse tensor image parameters in fiber tracking.

	Healthy control		Patient	
Tracts	Left	Right	Left	Right
Corticospinal tract				
Number of tract	15,462	13,499	12,492	10,657
Tract volume, mm^3^	15,227.3	12,501.3	9172.86	8228.45
Mean of FA	0.546	0.551	0.577	0.602
Mean of MD	0.784	0.729	0.792	0.784
Mean of AD	1.274	1.232	1.376	1.390
Somatosensory tract				
Number of tract	15,664	13,134	11,908	12,786
Tract volume, mm^3^	11,138.3	9106.83	2869.21	3307.94
Mean of FA	0.554	0.571	0.557	0.559
Mean of MD	0.725	0.730	0.791	0.896
Mean of AD	1.227	1.232	1.349	1.521
Acoustic radiation				
Number of tract	3569	2000	–	–
Tract volume, mm^3^	2029.44	3262.18	–	–
Mean of FA	0.515	0.438	–	–
Mean of MD	0.790	0.765	–	–
Mean of AD	1.309	1.169	–	–

**Figure 3:**
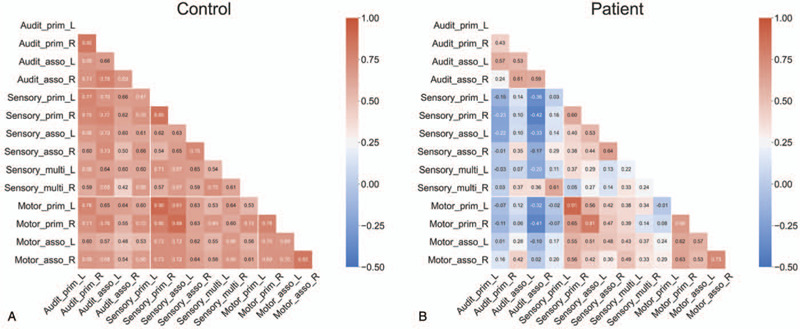
Matrix of functional connectivity in auditory, sensory, and motor networks in a healthy control (A) and the patient (B). Auditory_prim_L: the primary auditory cortex, left side; Auditory_prim_L: the primary auditory cortex, right side; Auditory_asso_L: the auditory association cortex, left side; Auditory_asso_R: the auditory association cortex, right side; Sensory_prim_L: the primary somatosensory cortex, left side; Sensory_prim_R: the primary somatosensory cortex, right side; Sensory_asso_L: the secondary somatosensory association cortex, left side; Sensory_asso_R: the secondary somatosensory association cortex, right side; Sensory_multi_L: the multimodal sensory association cortex, left side; Sensory_multi_R: the multimodal sensory association cortex, right side; Motor_supp_L: the motor supplementary cortex, left side; Motor_supp_R: the motor supplementary cortex, right side; Motor_prim_L: the primary motor cortex, left side; Motor_prim_R: the primary motor cortex, right side.

From rs-fMRI, a decreasing tendency in functional connectivity between bilateral auditory networks (primary auditory cortex, auditory association cortex) and bilateral motor networks (primary motor cortex, motor supplementary cortex) were shown in the patient (Fig. 3B) compared with a healthy control (Fig. 3A). In addition, a decreasing tendency in functional connectivity between bilateral auditory networks and bilateral sensory networks (primary somatosensory cortex, secondary association cortex, and multimodal sensory association cortex) was observed (Fig. 3). Furthermore, a decreasing tendency functional connectivity between bilateral primary auditory cortices and bilateral auditory association cortices was shown in the patient compared with a healthy control (Fig. 3).

## 3 Discussion

We used rs-fMRI data to investigate whether the functional connectivity between auditory and sensorimotor networks were altered in a patient with cortical deafness following bilateral hypertensive putaminal hemorrhage. To our knowledge, this is the first study to reveal that cortical alteration between auditory and sensorimotor networks in a patient with cortical deafness, motor, and sensory impairments after bilateral subcortical hemorrhagic stroke.

Cortical deafness was first reported by Wernicke and Friedlander in 1883 and refers to hearing loss caused by the central lesion without peripheral hearing problems.^[5]^ Both linguistic and nonlinguistic stimuli are affected.^[1]^ Typical brain lesions that cause cortical deafness are damage to bilateral Heschl gyri or acoustic radiations. Because acoustic radiations pass through the narrow space between the caudate nucleus and the internal capsule, several previous studies reported cortical deafness after bilateral putaminal lesions.^[2,3,6,7]^

Previous attempts had been made to evaluate changes in structural and functional connectivity associated with hearing loss through DTI and rs-fMRI. Lee et al^[8]^ reported that FA index on central auditory pathway was reduced in the sensorineural hearing loss group compared to the control group. Liu et al^[9]^ examined functional alterations in auditory, recognition, visual, and language networks in patients with unilateral sensorineural hearing loss. In addition, altered connectivity between auditory areas and parahippocampal, prefrontal, and occipital areas in tinnitus was reported by Maudoux et al.^[10]^

In our case, structural and functional disruptions of auditory networks might have interrupted reciprocal activations between auditory and motor networks. Our findings demonstrate the decrease in functional connectivity between auditory and motor related networks in a patient who suffered from motor deficits. These interruptions therefore may have caused imbalance and motor impairments even 6 months after stroke.

Compared with a healthy control, the decreased functional connectivity between auditory networks and somatosensory networks, especially multimodal sensory cortex, was shown in the patient with cortical deafness and sensory dysfunction. The primary auditory and auditory association cortices were functionally close linked to multisensory convergence, which has been proven as multimodal sensory cortex, at early stage of cortical auditory processing.^[11]^ Functional dysfunction in primary auditory and auditory association cortices due to disruption of acoustic radiation following subcortical hemorrhagic stroke might influence decreased connection of multimodal sensory cortex with auditory networks. Finally, in our case, complete hearing impairment persisted in 6 months after bilateral putaminal hemorrhagic stroke. It might be caused by structural disruption of acoustic radiation and functional reduction of connectivity in intra-auditory networks.

There was some limitation in this study. First, our DTI had 25 gradient directions. Improving the reliability of DTI measurements is needed to achieve high signal-to-noise (SNR) and to optimize DTI acquisition.^[12]^ Recent studies demonstrated that a large number of uniformly distributed gradient directions had been shown to reduce noise-induced rotational variance caused by the misalignment between the principal diffusion direction of the tensor and the gradient direction.^[13,14]^ However, Heiervang et al^[15]^ reported that the scan time was much shorter for DTI with 12 directions and the intersession variability was similar to DTI with 60 directions. This indicates that additional studies are needed to clarify the effect of number of gradient sampling directions on DTI reliability. Second, we provided rs-fMRI data between a patient and a healthy control from 2 different scanners. For comparison of rs-fMRI data between a patient and a healthy control, we analyzed temporal SNR (tSNR) of each scanner, which can be used to determine the SNR of fMRI time series.^[16,17]^ We confirmed that there was no significant difference in tSNR between 2 scanners using modified *t* test (*t* = -1.411, *P* = .097).

## 4 Conclusion

Our case report seems to suggest that functional alterations of spontaneous neuronal activity in auditory and sensorimotor networks would be related to motor and sensory impairments in the patient with cortical deafness after bilateral putaminal hemorrhagic stroke.

## Acknowledgments

The authors would like to express their gratitude to patient and family who agreed to publish.

## Author contributions

**Conceptualization:** Dae-Won Gwak.

**Data curation:** Dae-Won Gwak, Jin-Su Park, Eunji Kim.

**Investigation:** Jae-Eun Lee, Seung-Hwan Jung, Jae-Gyeong Jeong.

**Methodology:** Eunhee Park, Jin-Su Park, Eunji Kim, Min-Gu Kang, Ae-Ryoung Kim, Kyu-Yup Lee.

**Project administration:** Dae-Won Gwak, Min-Gu Kang, Ae-Ryoung Kim.

**Software:** Jin-Su Park, Eunji Kim.

**Supervision:** Tae-Du Jung.

**Visualization:** Eunhee Park, Jin-Su Park, Eunji Kim.

**Writing – original draft:** Dae-Won Gwak.

**Writing – review & editing:** Eunhee Park, Yongmin Chang.

## Supplementary Material

Supplemental Digital Content

## Supplementary Material

Supplemental Digital Content
