# Supplementary material for: Alterations of functional connectivity in auditory and sensorimotor neural networks: A case report in a patient with cortical deafness after bilateral putaminal hemorrhagic stroke
Source: Medicine (Baltimore). 2021 Jan 22;100(3):e24302. doi: 10.1097/MD.0000000000024302 (PMC7837815; doi:10.1097/MD.0000000000024302)
Supplement: Supplemental Digital Content [file medi-100-e24302-s001.docx]

| Hemisphere | Region of Interest | Center MNI coordinates (mm) | | | Volume | | Voxel |
| --- | --- | --- | --- | --- | --- | --- | --- |
|  |  | x | y | z | (mm^3^) | counts | |
| Left | Primary motor cortex | -27.4 | -17.2 | 59.8 | 1542.2 | 573 | |
|  | Primary somatosensory cortex | -29.6 | -29.0 | 58.8 | 1273.3 | 473 | |
|  | Hescle’s gyrus | -45.2 | -27.9 | 8.3 | 764.4 | 284 | |
|  | Anterior pons | -5.9 | -22.1 | -34.3 | 1047.1 | 389 | |
|  | Posterior pons | -4.5 | -35.3 | -31.8 | 820.9 | 305 | |
|  | Acoustic radiation | -21.2 | -34.5 | 6.4 | 1760.2 | 654 | |
| Right | Primary motor cortex | -31.3 | -16.8 | 58.4 | 1405 | 522 | |
|  | Primary somatosensory cortex | 30.1 | -32.3 | -31.8 | 1356.5 | 504 | |
|  | Hescle’s gyrus | 38.9 | -20.1 | 8.3 | 635.2 | 236 | |
|  | Anterior pons | 6.8 | -20.7 | -34.1 | 917.8 | 341 | |
|  | Posterior pons | 6.3 | -34.5 | -31.3 | 820.9 | 305 | |
|  | Acoustic radiation | 28.0 | -36.1 | 12.4 | 1294.6 | 481 | |

**Table S1.** Information of Region of Interests (ROIs) using Diffusion Tensor Tractography in a Patient with Bilateral Putaminal Hemorrhagic Stroke
