# Supplementary material for: Alterations of functional connectivity in auditory and sensorimotor neural networks: A case report in a patient with cortical deafness after bilateral putaminal hemorrhagic stroke
Source: Medicine (Baltimore). 2021 Jan 22;100(3):e24302. doi: 10.1097/MD.0000000000024302 (PMC7837815; doi:10.1097/MD.0000000000024302)
Supplement: Supplemental Digital Content [file medi-100-e24302-s002.docx]

**Table S2.** Information of Region of Interests (ROIs) using Resting-state Functional MRI in a Patient with Bilateral Putaminal Hemorrhagic Stroke

| Hemisphere | Region | MNI Coordinates (mm) | | |
| --- | --- | --- | --- | --- |
|  |  | x | y | z |
| Left | Primary auditory cortex | -46 | -21 | 10 |
|  | Auditory association cortex | -54 | -34 | 14 |
|  | Primary somatosensory cortex | -40 | -28 | 46 |
|  | Secondary somatosensory association cortex | -40 | -12 | 18 |
|  | Multimodal sensory association cortex | -52 | -32 | 32 |
|  | Primary motor cortex | -36 | -18 | 48 |
|  | Motor association cortex | -6 | -2 | 56 |
| Right | Primary auditory cortex | 46 | -20 | 10 |
|  | Auditory association cortex | 58 | -26 | 12 |
|  | Primary somatosensory cortex | 42 | -28 | 46 |
|  | Secondary somatosensory association cortex | 42 | 10 | 18 |
|  | Multimodal sensory association cortex | 52 | -34 | 34 |
|  | Primary motor cortex | 38 | -18 | 46 |
|  | Motor association cortex | 4 | -4 | 56 |
